# Molar incisor hypomineralization: Prevalence, severity and associated aetiological factors in children seeking dental care at Armed Forces Hospital Jazan, Saudi Arabia

**DOI:** 10.1016/j.sdentj.2024.06.003

**Published:** 2024-06-04

**Authors:** Mohammed Zameer, Syed Wali Peeran, Syed Nahid Basheer, Syed Ali Peeran, Gulam Anwar Naviwala, Sameen Badiujjama Birajdar

**Affiliations:** a Dental Department, Armed Forces Hospital, Abu Arish, Jazan, Saudi Arabia; b Department of Periodontics, Jazan University, Jazan, Saudi Arabia; c Division of Operative Dentistry,Department of Restorative Dental Sciences, Jazan University, Jazan, Saudi Arabia; d Olive Dental Clinic, Chennai, India; e Commissionerate of Health Services, Public Health Department, Mumbai, Maharashtra, India; f Sanjeevani Dental Clinic, Raichur, India

**Keywords:** Molar Incisor Hypomineralisation, Prevalence, Severity, Aetiology

## Abstract

**Background:**

The prevalence of molar incisor hypomineralisation (MIH) varies worldwide. In Saudi Arabia, data about this condition is limited to a few cities.

**Aim:**

To evaluate the prevalence, severity and associated aetiological factors of MIH in children seeking dental care in Armed Forces Hospital, Jazan, Saudi Arabia.

**Methodology:**

Participants were recruited by convenience sampling according to eligibility criteria. Diagnostic criteria used were according to the molar incisor hypomineralisation severity scoring system (MIH-SSS). Demographic data and past medical history were recorded using a carefully organised questionnaire, and MIH causal factors were evaluated.

**Results:**

A total of 1405 children participated in the study. Among the permanent first molars, mandibular teeth were more frequently affected by MIH than maxillary teeth. In the permanent central incisors group, maxillary teeth were more frequently involved than mandibular teeth, whereas lateral incisor was the least affected among the tooth types in all four quadrants. MIH had more frequently involved all four molars (66.1%), and two associated central incisors were found (31.3%). MIH in the incisors had mild to moderate severity, whereas molars presented with severe defects. Among prenatal factors, maternal anaemia and vitamin D deficiency, out-of-perinatal factors, caesarean delivery, low birth weight and perinatal jaundice, and early childhood tonsillitis and early childhood anaemia were the significant associated factors for MIH development.

**Conclusion:**

The prevalence of MIH was 8%, and maxillary incisors and mandibular first molars were frequently affected. Children with MIH showed prenatal, perinatal and postnatal aetiological factors involved in the development of MIH.

## 1 Introduction

Molar incisor hypomineralization (MIH) is a qualitative developmental enamel defect with a multifactorial aetiology, affecting one to four permanent first molars and permanent incisors. (Zameer and Birajdar, 2022) The colour of MIH defects may present as white, yellow, or brownish opacities. The severity of MIH defects varies among teeth, even in the same individual. The degree of defect opacity is related to the degree of porosity, and teeth will evolve to post-eruptive breakdown (PEB) when present with darker opacities. (Cabral et al., 2020, Zameer et al., 2020).

The condition has multiple negative implications that are significant for affected children and paediatric dentists. Hypersensitive teeth, rapid progression of dental caries, and aesthetic repercussions are negative implications in affected children. As for paediatric dentists, the implications include complexity in treatment planning, poor prognoses of the restorations, and difficulty in achieving pain management during treatment (Al-Othaibi et al., 2019).

MIH with multifactorial aetiology has been explored. Environmental disturbances (during the prenatal, perinatal, and postnatal periods) and genetic factors are associated with the causative mechanism of MIH (Alhowaish et al., 2021). The timing of occurrence, duration and strength of causal factors are accountable for a spectrum of defects. (Lygidakis et al., 2022) An understanding of causal factors associated with MIH is important to the early identification of high-risk children and formulation of prevention strategies that can reduce negative implications.

The prevalence of MIH varies by region and birth cohort. In the Arabian Peninsula in general and in Saudi Arabia in particular, the prevalence of MIH has been rarely studied. The prevalence of MIH in Saudi Arabia ranges from 8.6 % to 40.7 %. (Al-Hammad et al., 2018, Alhowaish et al., 2021, Allazzam et al., 2014; ijar & Hammad, 2017; Rizk et al., 2018) Variation among the studies can be explained by the differences in sampling technique, age groups and examination variability. Further research is needed to elucidate the possibility of other reasons for this variation. Moreover, scientific baseline data regarding the prevalence of MIH in the Jazan region is lacking. Therefore, the present study aimed to evaluate the prevalence and severity of MIH and its causal factors in children treated at the Armed Forces Hospital, Jazan, Saudi Arabia.

## 2 Methodology

Ethical Approval: The research methodology of this study was approved by the ethical committee of Armed Forces Hospital, Jazan, Saudi Arabia.

### 2.1 Study design

An observational cross-sectional study was conducted on Saudi children from military families at the paediatric dental clinic of Armed Forces Hospital, Jazan, Saudi Arabia. The participants were selected by convenience sampling (n = 1405) based on the eligibility criteria from June 2022 to May 2023. Informed consent was obtained from the parents of the selected children.

Eligibility Criteria.

i) Inclusion Criteria.

ii) Consent to participate.

iii) Age of 8–12 years.

iv) Fully erupted index teeth (permanent first molars and incisors).

Exclusion Criteria.

i. Orthodontic appliance.
ii. Teeth with other types of enamel defects.
iii. Extensive destruction of teeth due to dental caries.

Diagnostic Criteria: The molar incisor hypomineralisation severity scoring system (MIH-SSS), a recently developed criterion (Cabral et al., 2020) was adopted for assessing MIH defects and classifying severity according to clinical characteristics.

### 2.2 Calibration of the Examiner

All clinical examinations were carried out by a single well-trained calibrated Paediatric dentist. The examiner was trained to diagnose and differentiate MIH and other development defects of the enamel, and a calibration exercise utilizing the clinical photographs of 30 patients (15 cases of MIH and 15 with other development defects) was implemented. The same procedure was repeated one month later, and the reproducibility index was calculated using Cohen’s Kappa coefficient. The intra-examiner agreement was 0.96 (excellent).

### 2.3 Setting and data Collection

Demographic details were recorded for each study participant. The index teeth in both jaws were cleaned using prophylactic paste. Clinical examination was performed at the hospital’s paediatric dental clinic. The hardness of the affected dentin was checked with a plain dental intra-oral mirror and an explorer. Tooth surfaces were examined wet by a calibrated examiner following the MIH-SSS. Defects with > 1 mm diameters were recorded. When two or more defects were observed in the same tooth surface, the more severe defect was recorded.

Questionnaire: Causal factors for MIH were identified by analysing the scientific literature, and then a questionnaire was constructed, which was translated into the vernacular language and translated back to English (Appendix 1). The parents of the study participants were interviewed either face to face in the clinic or over the phone. Collected data were tabulated for analysis.

### 2.4 Statistical analysis

Descriptive and inferential statistical analyses were carried out. The results of continuous measurements were presented as mean ± SD, and results on categorical measurement were presented in number (%). The level of significance was set at p = 0.05, and any value less than or equal to 0.05 was statistically significant. The significance of study parameters on a categorial scale was assessed through chi-square analysis. The influences of medical health variables on MIH were investigated using logistic regression analysis. The independent variables were the questions asked in the medical questionnaire, whereas the dependent variable was MIH. The statistical software IBM SPSS Statistics 20.0 (IBM Corporation, Armonk, NY, USA) was used for data analysis, and Microsoft Word and Excel were used to generate tables.

## 3 Results

Of the 1478 children approached to participate in the study, only 1405 children fulfilled the inclusion criteria and agreed to participate. The demographic characteristics of the participants and prevalence of MIH are shown in Table 1. The prevalence of MIH in our study participants was 8 % (n = 112). The 10-year age group showed the highest sample population (26.3 %), whereas the 12-year age group represented the smallest sample size (8.6 %).

**Table 1:** Demographic characteristics and descriptive statistics of the study participants (N = 1405).

Variables	Sub-groups	n	%
**Gender**	**Male**	714	50.8
	**Female**	691	49.2
**Age** **(in years)**	**8**	241	17.2
	**9**	364	25.9
	**10**	369	26.3
	**11**	310	22.1
	**12**	121	8.6
**Age (Mean ± SD)**		9.79 ± 1.211	
**Prevalence of Molar Incisor Hypo mineralization**	**MIH + ve**	112	8.0
	**MIH −ve**	1293	92.0

MIH + ve: affected with molar incisor Hypomineralisation, MIH −ve: not affected with molar incisor Hypomineralisation

The distribution patterns of the affected first permanent molars and permanent incisors for each study participant are shown in Table 2. The association among the MIH-affected children, who presented with defects in 706 teeth (374 M and 332 incisors), showed that MIH most frequently involved all four molars (66.1 %) and two central incisors (31.3 %).

**Table 2:** Distribution patterns of the affected first permanent molars and permanent incisors in study participants (N = 112).

**Variables**	**Sub-groups**	**n**	**%**
**Quadrants affected**	**One quadrant affected**	4	3.6
	**Two quadrants affected**	7	6.3
	**Three quadrants affected**	14	12.5
	**Four quadrants affected**	87	77.7
**First permanent molars**	**One Molar affected**	8	7.1
	**Two molars affected**	20	17.9
	**Three molars affected**	10	8.9
	**Four molars affected**	74	66.1
**Permanent incisors**	**No Incisor affected**	10	8.9
	**One incisor affected**	11	9.8
	**Two incisors affected**	35	31.3
	**Three incisors affected**	12	10.7
	**Four incisors affected**	25	22.3
	**Five incisors affected**	7	6.3
	**Six incisors affected**	8	7.1
	**Eight incisors affected**	4	3.6

Table 3 illustrates the multivariate binary logistic regression analysis of the medical health variables in children who were affected by MIH (+ve) and those who were not (−ve). A variable was considered significant when the p-value was < 0.05. The results showed maternal anaemia, maternal vitamin D deficiency, caesarean delivery, low birth weight, perinatal jaundice, tonsillitis, and anaemia were significant predictors of MIH (p < 0.05).

**Table 3:** Comparison of the presence or absence of different variables among both the groups using the chi-square test.

Medical health variables	Availability	MIH + ve N (%)	MIH −ve N (%)	Odd’s Ratio	Confidence Interval	P-value
Maternal Anaemia	Present	17(15.2)	58(4.5)	3.810	2.12–6.80	<0.001**
	Absent	95(84.8)	1235(95.5)			
Maternal Vitamin D deficiency	Present	11(9.8)	31(2.4)	4.343	2.16–9.08	<0.001**
	Absent	101(90.2)	1262(97.6)			
Maternal Hypocalcaemia	Present	6(5.4)	134(10.4)	0.490	0.211–1.136	0.090
	Absent	106(94.6)	1159(89.6)			
Perinatal Jaundice	Present	19(17.0)	54(4.2)	4.688	2.66–8.23	<0.001**
	Absent	93(83.0)	1239(95.8)			
Anaemia	Present	12(10.7)	100(89.3)	3.963	2.00–7.82	<0.001**
	Absent	38(2.9)	1255(97.1)			
Preterm Birth	Present	10(8.9)	136(10.5)	0.834	0.42–1.63	0.597
	Absent	102(91.1)	1157(89.5)			
Low birth weight	Present	8(7.1)	184(14.2)	0.464	0.22–0.96	0.036*
	Absent	184(92.9)	1109(85.8)			
Tonsillitis	Present	9(8.0)	245(18.9)	0.374	0.18–0.74	0.004*
	Absent	103(92.0)	1151(81.9)			
Caesarean Delivery	Present	7(6.2)	163(12.6)	0.462	0.21–1.01	0.048*
	Absent	105(93.8)	1130(87.4)			
GIT problems	Present	4(3.6)	73(5.6)	0.619	0.22–1.72	0.355
	Absent	108(96.4)	1220(94.4)			
VitaminD Deficiency	Present	2(1.8)	33(2.6)	0.694	0.16–2.93	0.618
	Absent	110(98.2)	1260(97.4)			
Feeding difficulty	Present	2(1.8)	16(1.2)	1.451	0.32–6.39	0.621
	Absent	110(98.2)	1277(98.8)			
Frequent antibiotic intake	Present	3(2.7)	98(7.6)	0.336	0.10–1.07	0.054
	Absent	109(97.3)	1195(92.4)			
Cerebral palsy	Present	1(0.9)	5(0.4)	2.321	0.26–20.03	0.431
	Absent	111(99.1)	1288(99.6)			

p < 0.05 − Significant*, p < 0.001 − Highly significant**,

MIH + ve: affected with molar incisor Hypomineralisation, MIH −ve: not affected with molar incisor Hypomineralisation.

Table 4 shows the distribution of defects according to the MIH-SSS. Overall, MIH in incisors had mild to moderate severity, and molars presented with severe defects, which were mostly PEBs exposing hard dentin. Among the permanent first molars, mandibular teeth were more frequently affected with MIH (#36–92.0/#46–82.1 %) than maxillary teeth (#16–82.1/#26–77.7 %). In the permanent central incisors group, maxillary teeth were more frequently involved (#11–68.7/#21–75.0 %) than mandibular teeth (#31–25.0/#41–20.5 %), whereas laterals were the least affected among the tooth types in all four quadrants.

**Table 4:** Distribution of MIH defects according to MIH severity scoring system.

#		Mild		Moderate		Severe					=
	Sound	W OP	Y OP	PEB W	PEBY	PEBH	PEBS	SAR	UAR	EXT	
**16**	20 (17.9 %)	3 (2.7 %)	7 (6.3 %)	10 (8.9 %)	14 (12.5 %)	30 (26.8 %)	13 (11.6 %)	7 (6.3 %)	0 (0.0 %)	8 (7.1 %)	112
**26**	25 (22.3)	3 (2.7 %)	3 (2.7 %)	11 (9.8 %)	23 (20.5 %)	30 (26.8 %)	9 (8.0 %)	4 (3.6 %)	0 (0.0 %)	4 (3.6 %)	112
36	9 (8.0 %)	2 (1.8 %)	4 (3.6 %)	7 (6.3 %)	9 (8.0 %)	42 (37.5 %)	18 (16.1 %)	13 (11.6 %)	6 (5.4 %)	2 (1.8 %)	112
**46**	20 (17.9 %)	1 (0.9 %)	7 (6.3 %)	3 (2.7 %)	17 (15.2 %)	29 (25.9 %)	10 (8.9 %)	6 (5.4 %)	12 (10.7 %)	7 (6.3 %)	112
**11**	35 (31.3 %)	13 (11.6 %)	43 (38.4 %)	5 (4.5 %)	14 (12.5 %)	2 (1.8 %)	0(0.0 %)	0 (0.0 %)	0 (0.0 %)	0 (0.0 %)	112
**12**	70 (62.5 %)	21 (18.8 %)	10 (8.9 %)	7 (6.3 %)	4 (3.6 %)	0 (0.0 %)	0 (0.0 %)	0 (0.0 %)	0 (0.0 %)	0 (0.0 %)	112
**21**	28 (25.0 %)	29 (25.9 %)	35 (31.3 %)	9 (8.0 %)	11 (9.8 %)	0 (0.0 %)	0 (0.0 %)	0 (0.0 %)	0 (0.0 %)	0 (0.0 %)	112
**22**	74 (66.1 %)	26 (23.2 %)	6 (5.4 %)	4 (3.6 %)	2 (1.8 %)	0 (0.0 %)	0 (0.0 %)	0 (0.0 %)	0 (0.0 %)	0 (0.0 %)	112
**31**	84 (75.0 %)	22 (19.6 %)	3 (2.7 %)	0 (0.0 %)	3 (2.7 %)	0 (0.0 %)	0 (0.0 %)	0 (0.0 %)	0 (0.0 %)	0 (0.0 %)	112
**32**	96 (85.7 %)	10 (8.9 %)	4 (3.6 %)	0 (0.0 %)	2 (1.8 %)	0 (0.0 %)	0 (0.0 %)	0 (0.0 %)	0 (0.0 %)	0 (0.0 %)	112
**41**	89 (79.5 %)	19 (17.0 %)	1 (0.9 %)	0 (0.0 %)	3 (2.7 %)	0 (0.0 %)	0 (0.0 %)	0 (0.0 %)	0 (0.0 %)	0 (0.0 %)	112
**42**	97 (86.6 %)	9 (8.0 %)	2 (1.8 %)	0 (0.0 %)	4 (3.6 %)	0 (0.0 %)	0 (0.0 %)	0 (0.0 %)	0 (0.0 %)	0 (0.0 %)	112
**=**	647	158	125	56	106	133	50	30	18	21	

# − Tooth number, = − Total, W-White, Y-Yellow, OP-Opacity, PEB-Post eruptive breakdown, H-Hard dentin exposed, S-Soft dentin exposed, SAR/UAR-Satisfactory and Unsatisfactory atypical restorations respectively, EXT-Extraction due to MIH.

## 4 Discussion

The baseline prevalence of MIH in Jazan was 8 %, and 77.7 % of affected individuals were diagnosed with MIH in all four quadrants of their dentition. The study edifies paediatric dentists and raises awareness about this condition to enable early diagnosis and the implementation of appropriate measures. The findings differ from those of previous studies in Saudi Arabia (Al-Hammad et al., 2018, Alhowaish et al., 2021, Almuallem et al., 2022, ijar, & Hammad, N. , 2017, Rizk et al., 2018), except a study in Jeddah, which reported a similar rate of 8.6 % (Allazzam et al., 2014). Differences in sampling technique, age groups, and examination methods can be the reason for variations among the studies.

For research protocol standardization, experts advise including a minimum of 300 children in the calculation of MIH prevalence. The Jeddah study had a smaller (〈3 0 0) sample size (Allazzam et al., 2014). Our study had a sample size that was nearly fivefold. The age selected for our study was 8–12 years, when first permanent molars and incisors usually erupt. The present study was the first to examine MIH prevalence in the paediatric population in Jazan, Saudi Arabia, specifically focusing on Saudi children from military families.

In contrast to various proposed evaluation criteria for MIH, such as modified developmental defects of enamel and EAPD-criteria, MIH-SSS comprehensively records the characteristics of MIH defects and their severity based on their clinical characteristics. This new scoring system specifies the extent of PEB and whether it is restricted to the enamel or exposes soft or hard dentin, providing insight into how opacity transforms into a severe defect; the colour of opacity ranges from white or creamy to yellow or brown (Cabral et al., 2020). The detection criteria (MIH index vs MIH-SSS) for MIH were effective in diagnosing MIH and its characteristics. However, MIH-SSS is more comprehensive and comparatively more suitable for interpreting the severity of MIH and can be applied in a shorter average time. Another crucial factor of MIH-SSS is that it records only active atypical caries lesions; when they remineralize, they are categorised as PEB (Mendonça et al., 2023). Hence, this system was adopted in the present study.

Most of the teeth with MIH were permanent first molars. This result supported a previous finding that developing teeth are likely to be affected by MIH (Wogelius et al., 2008). However, children had different number of affected teeth or had the same severity of the defect, suggesting a potential differential genetic influence or environmental factors that may disturb the tooth development; meanwhile, the corresponding contralateral tooth may be unaffected. (Vieira & Manton, 2019).

The present study showed that the children commonly had four affected molars and two central incisors, similar to a previous study (Lygidakis et al., 2008a). The molars had more severe defects, and the highest prevalence of PEB was obtained in the molars. The probable reason was the old age range of children in the study; the affected molars were subjected to vigorous masticatory forces over an extended period. The prevalence of PEB increases with age (Bagattoni et al., 2022; Wogelius et al., 2008).

Regarding the possible predisposing factors for MIH, we observed a significant association between environmental disturbances during the prenatal, perinatal and postnatal periods and MIH. Maternal vitamin D deficiency, maternal anaemia, caesarean delivery, perinatal jaundice, early childhood anaemia, and tonsillitis were significant predictors of MIH. The findings of the present study are in contrast with the earlier studies from Saudi Arabia (Alhowaish et al., 2021, Allazzam et al., 2014, Almuallem et al., 2022) and an earlier systematic review (Silva et al., 2016) that demonstrated no significant association between prenatal factors and MIH. It reports substantial variability in the terminologies used to describe maternal illnesses during gestation. However, looking at the potential significance of prenatal disturbances, two recent systematic reviews found a positive correlation with MIH (Fatturi et al., 2019, Juárez-López et al., 2023). According to the findings, MIH is 40 % more likely to develop in children when mothers have health issues during pregnancy. The window period for tooth enamel maturation that can be affected by defects corresponds to the last trimester of pregnancy to the early three years of the child’s life. During this gestational period, any disease could affect the amelogenesis process and lead to MIH (Butera et al., 2021, Juárez-López et al., 2023).

In our study, we found that maternal vitamin D deficiency is a significant predisposing factor for the development of MIH in their children. This finding is consistent with previous study that showed an increased risk of MIH in the children of mothers who had vitamin D deficiency during pregnancy (Rai et al., 2018). According to research, taking high-dose vitamin D supplements during pregnancy can decrease the risk of enamel defects in children by approximately 50 %. (Nørrisgaard et al., 2019). Another significant prenatal factor in this study related to MIH is maternal anaemia. The risk of MIH was higher for children of mothers who had anaemia during pregnancy compared to those who did not (Mariam et al., 2022, Thakur et al., 2020) A recent systematic review reaffirmed this association (Wilmer et al., 2021).

Caesarean delivery was found to be a significant predictor for the development of MIH. An earlier study found that caesarean section deliveries can induce perinatal hypoxia, leading to enamel defects in children (Garot et al., 2022). Additionally, low birth weight was associated with MIH, and this association can be attributed to impaired enamel mineralisation or nutrient deficiencies during amelogenesis (Noor Mohamed et al., 2021, Wu et al., 2020).

A positive association was seen between perinatal jaundice and MIH. Similar findings have been published in the literature (Alhowaish et al., 2021, Bagattoni et al., 2022, Mariam et al., 2022, Thakur et al., 2020). Depending on the severity and duration of perinatal jaundice, many phenotypes of tooth discoloration through bilirubin activity are evident. The green discoloration was the most common, followed by brown, yellow and grey, and the degree of discoloration is relative to level of serum bilirubin (Park et al., 2017). Blood-derived albumin binds with the developing enamel and disrupts its mineralisation (Williams et al., 2020). Our present study is one of the few studies that found a significant association between perinatal jaundice and MIH. Future research can focus on investigating the influence of serum bilirubin during perinatal jaundice in the aetiopathogenesis of MIH.

The present study showed that tonsillitis is a predictor of MIH, consistent with the previous study (Né et al., 2022). However, the evidence for tonsillitis as a causative factor is exceptionally low, and high heterogeneity was found in studies on respiratory diseases as the cause of MIH. A recent study from Saudi Arabia showed a significant association between tonsillitis and MIH (Almuallem et al., 2022). Early childhood anaemia is rarely considered in earlier studies of MIH, with only one study mentioning it as an associated factor (Lygidakis et al., 2008b). However, an association was found between anaemia and MIH. Other studies have explored enamel defects in patients with sickle cell anaemia, showing a high prevalence of developmental defects (Lopes et al., 2018) Further research is needed to analyse the link between anaemia and MIH.

The current study has certain limitations based on its methodology. This study is limited to Saudi children from military families of Jazan province; therefore, the results may not be extrapolated across the country. However, this study will provide a novel perspective regarding the situation of MIH in Jazan province and Saudi Arabia as a whole. Moreover, the information gathered through interviews with parents may have bias because the respondents would not be able to recollect past information completely. Lastly, this retrospective study only established the possible association of the condition rather than the cause and effect. Future prospective investigations considering associated causal factors could enhance understanding of the aetiology of MIH.

## 5 Conclusion

The prevalence of MIH was 8 %, and maxillary incisors and mandibular first molars being more frequently affected. All four molars presented severe MIH, and PEB that exposes hard dentin was the most common defect. Children with MIH showed prenatal, perinatal and postnatal aetiological factors involved in the development of MIH.

## Appendix -1

**Questionnaire**.

Age?

Gender?

Place of residence?

Place of birth?

Was the mother suffering from anaemia during pregnancy?

Has the mother had any deficiency of vitamin D during pregnancy?

Has the mother had any deficiency of calcium during pregnancy?

Was your child diagnosed with newborn jaundice requiring treatment under light?

Did your child suffer from anaemia at or before the age of three?

Was your child full-term/pre-term?

Has your child had a low birth weight?

Did your child have tonsillitis at or before the age of three?

Was the child delivery normal/Caesarean?

Did your child have GIT problems at or before the age of three?

Did your child suffer Vitamin D deficiency at or before the age of three?

Has your child had feeding difficulty?

Did your child receive frequent antibiotics at or before the age of three?

Was your child diagnosed with cerebral palsy at or before the age of three?
